# Multiple Control Strategies for Prevention of Avian Influenza Pandemic

**DOI:** 10.1155/2014/949718

**Published:** 2014-02-24

**Authors:** Roman Ullah, Gul Zaman, Saeed Islam

**Affiliations:** ^1^Department of Mathematics, Bacha Khan University, Charsadda 24420, Pakistan; ^2^Department of Mathematics, Abdul Wali Khan University Mardan, Mardan 23200, Pakistan; ^3^Department of Mathematics, University of Malakand, Chakdara 23101, Pakistan

## Abstract

We present the prevention of avian influenza pandemic by adjusting multiple control functions in the human-to-human transmittable avian influenza model. First we show the existence of the optimal control problem; then by using both analytical and numerical techniques, we investigate the cost-effective control effects for the prevention of transmission of disease. To do this, we use three control functions, the effort to reduce the number of contacts with human infected with mutant avian influenza, the antiviral treatment of infected individuals, and the effort to reduce the number of infected birds. We completely characterized the optimal control and compute numerical solution of the optimality system by using an iterative method.

## 1. Introduction

Highly pathogenic avian influenza is a zoonotic disease caused by H5N1 virus and it has a devastating impact on poultry causing above 90 percent mortality within 48 hours of infection [1]. The disease also spreads to humans through the direct contact with the infected poultry. Despite all advances of medical, avian influenza still poses a significant threat to health and economy of the society. The laboratory confirmed that the fatality rate in human case is about 60 percent [2].

Several reports have so far been made of possible coinfection of humans with an H5N1 strain and a human strain [3, 4]. One of the coinfection reports is of a Turkish family cluster. In late December 2005 and early January 2006, a cluster of 8 confirmed highly pathogenic avian influenza (H5N1) cases was detected in Dogubayazit district in eastern Turkey [5]. All confirmed case patients were hospitalized after onset of symptoms. Four of them were died and the other four recovered. Another cluster of 8 cases of HPAI (H5N1) was detected in the village of Kubu Sambilang, Samatra, Indonesia, in April-May 2006 [6]. Only one of them recovered and the remaining seven members died. Though the human-to-human transmission of the disease is rare, its potential to change into an extremely high virulent human-to-human transmittable pandemic strain is the real danger to human health. Due to this threat, some international organizations have developed and began to implement different strategies (biosecurity, isolation, and antiviral treatment) for forestalling the onset of a pandemic. It has been estimated that a future avian influenza pandemic could cause hundreds of billions of dolors due to lost productivity, associated medical policies, and prevention policies.

Mathematical models have been widely used to evaluate the effects of control measures of avian influenza [7–9]. In 2007, Iwami et al. [10] proposed a mathematical model of the spread of avian influenza from bird population to human population. They discussed that to minimize the spread of the disease in human population, we must take the measures for the infected human with bird flu to quarantine when mutant bird flu has already occurred. Lee et al. [11] show that the optimal control strategies that rely on the use of antiviral treatment or isolation strategies can reduce the number of clinical cases; therein they emphasize isolation strategies in mitigation of pandemic of influenza particularly when the access to antiviral resources is limited. Later on Ullah et al. [12] proposed the control strategies where they focus on minimizing the impact of influenza by minimizing the vaccine wanning.

In this paper, we focus on identifying the optimal control strategies for the model developed in [13]; these controls minimize the impact of avian influenza by isolating the infected individuals, the judicious use of drug supply, and eliminating the infected birds. Recommended medicines like oseltamivir or zanamivir must be taken within two days after the appearance of symptoms. The clinically ill and infectious individuals can be isolated by reasonably effective ways to reduce the transmission of disease, like to educate them to cover their sneeze and cough, not to spit openly, and to avoid the closed contacts with others, sanitizing the rooms or equipments occupied by the patients. The diagnosed infected birds should be culled within a few hours and all the infected dead birds and other contaminated objects (faeces, blood, and feathers) must be destroyed properly (burying, incineration) as soon as possible. We can get the most effective results if we use the isolation, treatment of clinically infectious humans, and elimination of infected birds concurrently.

## 2. Model Frame Work

In this section, the compartmental model that divides the human and birds populations into two different classes presents the optimal control problem for the transmission dynamics of avian influenza. Our main aim is to show that it is possible to implement the time-dependent antiavian influenza control techniques while minimizing the cost of such measures. In order to do this, we introduce three time-dependent control functions *u*_1_, *u*_2_, and *u*_3_. The control *u*_1_ represents the effort to reduce the number of contacts with human infected by mutant avian influenza, *u*_2_ represents the fraction of clinically infectious cases treated with antiviral per unit of time, and *u*_3_ is the effort to reduce the number of infected birds. Note that the controls are fully effective when *u*_*i*_ = 1 for *i* = 1,2, 3, while there is no control if *u*_*i*_ = 0. We divide the total human population at time *t* denoted by *N*_*h*_ into five distinct epidemiological subclasses which are susceptible *X*_*h*_, exposed *E*_*h*_, clinically ill and infectious *I*_*h*_, treated *T*_*h*_, and recovered *R*_*h*_ and the birds population *N*_*b*_ into three distinct subclasses which are susceptible *X*_*b*_, exposed *E*_*b*_, and infected *I*_*b*_. Taking into account the assumptions above, the dynamics of the control problem is given by

(1) $$\begin{array}{c} \frac{dX_{h}}{dt}=\Lambda -\mu_{h}X_{h}-\left(1-u_{1}\right)\alpha_{1}X_{h}I_{h}-\alpha_{2}X_{h}I_{b}+\epsilon_{h}R_{h},\\ \frac{dE_{h}}{dt}=\left(1-u_{1}\right)\alpha_{1}X_{h}I_{h}+\alpha_{2}X_{h}I_{b}-\left(\mu_{h}+\phi_{h}\right)E_{h},\\ \frac{dI_{h}}{dt}=\phi_{h}E_{h}-\left(\rho_{h}+\beta_{h}+\mu_{h}+u_{2}\right)I_{h},\\ \frac{dT_{h}}{dt}=\rho_{h}I_{h}-\left(\gamma_{h}+\mu_{h}\right)T_{h},\\ \frac{dR_{h}}{dt}=\gamma_{h}T_{h}+u_{2}I_{h}-\left(\mu_{h}+\epsilon_{h}\right)R_{h},\\ \frac{dX_{b}}{dt}=\pi -\mu_{b}X_{b}-\left(1-u_{3}\right)\alpha_{3}X_{b}I_{b},\\ \frac{dE_{b}}{dt}=\left(1-u_{3}\right)\alpha_{3}X_{b}I_{b}-\left(\mu_{b}+\phi_{b}\right)E_{b},\\ \frac{dI_{b}}{dt}=\phi_{b}E_{b}-\left(\beta_{b}+\mu_{b}\right)I_{b}, \end{array}$$

with the initial conditions

(2) $$\begin{array}{c} X_{h}\left(0\right)\geq 0,\;E_{h}\left(0\right)\geq 0,\;I_{h}\left(0\right)\geq 0,\\ T_{h}\left(0\right)\geq 0,\;R_{h}\left(0\right)\geq 0,\;X_{b}\left(0\right)\geq 0,\\ E_{b}\left(0\right)\geq 0,\;I_{b}\left(0\right)\geq 0. \end{array}$$

The human population is recruited at a constant birth rate Λ, *ϵ*_*h*_ is the rate of immunity loss, *ϕ*_*h*_ is the progression rate from *E*_*h*_ to *I*_*h*_, *ρ*_*h*_ represents the treatment rate of human, *μ*_*h*_ and *μ*_*b*_ are the natural death rates of humans and birds, respectively, *β*_*h*_ and *β*_*b*_ are disease induced death rates in humans and birds, respectively, *π* represents recruitment rate of birds population, *γ*_*h*_ shows recovery due to treatment, *ϕ*_*b*_ is progression rate of birds from *E*_*b*_ to *I*_*b*_, and *α*_1_, *α*_2_, and *α*_3_ are effective contact rates between *X*_*h*_ and *I*_*h*_, *X*_*h*_ and *I*_*b*_, and *X*_*b*_ and *I*_*b*_, respectively.

The objective of our control problem is to minimize the number of clinically infectious humans and infected birds and the cost of implementing the control by using possible minimal control variables *u*_*i*_(*t*) for *i* = 1,2, 3. We use the Lebesgue measurable control and define our objective functional as

(3) J(u1,u2,u3)  =∫0tend(A1Ih+A2Ib+12(C1u12+C2u22+C3u32))dt,

where *A*_1_ and *A*_2_ are positive constants to keep a balance in the size of *I*_*h*_ and *I*_*b*_. The square of the control variables reflects the severity of the side effects of the controls. *C*_1_, *C*_2_, and *C*_3_ are positive weight parameters such that 0 < *C*_1_ < Λ/*μ*_*h*_, 0 < *C*_2_ < Λ/*μ*_*h*_, and 0 < *C*_3_ < *π*/*μ*_*b*_. The objective of the optimal control problem is to seek optimal control functions (*u*_1_*(*t*), *u*_2_*(*t*), *u*_3_*(*t*)) such that

(4) $$\begin{array}{c} J\left(u_{1}^{*},u_{2}^{*},u_{3}^{*}\right)=\underset{(u_{1},u_{2},u_{3})\in U}{\min}\left\{J\left(u_{1},u_{2},u_{3}\right)\;|\;\left(u_{1},u_{2},u_{3}\right)\in U\right\}, \end{array}$$

where the control set is defined as

(5) U={u=(u1,u2,u3) ∣ ui  is  Lebesgue  measurable on⁡ [0,1],0≤ui(t)≤1, t∈[0,tend], for  i=1,2,3},

subject to the system (1) with appropriate initial conditions. Pontryagin's maximum principle [14] is used to solve this optimal control problem and the derivation of the necessary conditions. First we prove the existence of an optimal control for the system (1) and then derive the optimality system.

## 3. Existence of Control Problem

In this section, we consider the control system (1) with initial conditions at *t* = 0 to show the existence of the control problem. Note that, for the bounded Lebesgue measurable controls and nonnegative initial conditions, nonnegative bounded solutions to the state system exist (see [15]). To prove the existence of the solution of system (1), we can write

(6) $$\begin{array}{c} Y_{t}=BY+F\left(Y\right), \end{array}$$

where

(7) Y=[XhEhIhThRhXbEbIb],  F(Y)=[Λ−(1−u1)α1XhIh−α2XhIb(1−u1)α1XhIh+α2XhIb000π−(1−u3)α3XbIb(1−u3)α3XbIb0],B=[−μh000ϵh0000−μh−ϕh0000000ϕh−ρh−βh−μh−u20000000ρh−γh−μh0000000γh+u2−μh−ϵh000μb0000000000000−μb−ϕb0000000ϕb−βb−μb],

where *Y*_*t*_ denotes derivative of *Y* with respect to time *t*. Equation (3) is a nonlinear system with a bounded coefficient. We set

(8) $$\begin{array}{c} D\left(Y\right)=BY+F\left(Y\right). \end{array}$$

Now,

(9) F(Y1)−F(Y2)=[(1−u1)α1(Xh2Ih2−Xh1Ih1)+α2(Xh2Ib2−Xh1Ib1)−(1−u1)α1(Xh2Ih2−Xh1Ih1)−α2(Xh2Ib2−Xh1Ib1)000(1−u3)α3(Xb2Ib2−Xb1Ib1)−(1−u3)α3(Xb2Ib2−Xb1Ib1)0],|F(Y1)−F(Y2)| =|(1−u1)α1(Xh2Ih2−Xh1Ih1)+α2(Xh2Ib2−Xh1Ib1)|  +|(1−u1)α1(Xh2Ih2−Xh1Ih1)+α2(Xh2Ib2−Xh1Ib1)|  +|(1−u3)α3(Xb2Ib2−Xb1Ib1)|  +|(1−u3)α3(Xb2Ib2−Xb1Ib1)| ≤M[|Xh2−Xh1|+|Ih2−Ih1|+|Xb2−Xb1|+|Ib2−Ib1|],

where

(10) M=max⁡{2[(1−u1)α1Λμh+α2πμb],2[(1−u3)α3πμb+α2Λμh]}.

Here the constant *M* is independent of the state variables *X*_*h*_, *I*_*h*_, *R*_*h*_, *X*_*b*_, and *I*_*b*_. Also we get |*D*(*Y*_1_) − *D*(*Y*_2_)| ≤ *V*|*Y*_1_ − *Y*_2_|, where *V* = max⁡{*M*, ||*B*||}, which shows that *D* is uniformly Lipschitz continuous; hence by definition, the solution of the system exists (see [15]).

Let us go back to the optimal control problem (1)–(3). In order to find an optimal solution, first we should find the Lagrangian and Hamiltonian for the optimal control problem. The Lagrangian is

(11) $$\begin{array}{c} L=A_{1}I_{h}+A_{2}I_{b}+\frac{1}{2}\left(C_{1}u_{1}^{2}+C_{2}u_{2}^{2}+C_{3}u_{3}^{2}\right). \end{array}$$

We seek for the minimal value of the Lagrangian. To do this, we define the Hamiltonian *H* for the control problem, where *λ*_*i*_, *i* = 1,2,…, 8, are the adjoint variables:

(12) H=A1Ih+A2Ib+12(C1u12+C2u22+C3u32) +λ1[Λ−μhXh−(1−u1)α1XhIh−α2XhIb+ϵhRh] +λ2[(1−u1)α1XhIh+α2XhIb−(μh+ϕh)Eh] +λ3[ϕhEh−(ρh+βh+μh+u2)Ih] +λ4[ρhIh−(γh+μh)Th] +λ5[γhTh+u2Ih−(μh+ϵh)Rh] +λ6[π−μbXb−(1−u3)α3XbIb] +λ7[(1−u3)α3XbIb−(μb+ϕb)Eb] +λ8[ϕbEb−(βb+μb)Ib].

For the existence of the control system (1), we state and prove the following theorem.

**Theorem 1**

There exists an optimal control u* = (*u*_1_*, *u*_2_*, *u*_3_*) ∈ *U* such that

(13) $$\begin{array}{c} J\left(u_{1}^{*},u_{2}^{*},u_{3}^{*}\right)=\underset{(u_{1},u_{2},u_{3})\in U}{\min}\;J\left(u_{1},u_{2},u_{3}\right) \end{array}$$

subject to the control system (1) with the initial conditions (2).

**Proof**

To prove the existence of an optimal control, we use the result in [16–18]. Note that the control and the state variables are non-negative values. In this minimizing problem, the necessary convexity of the objective functional in *u*_1_, *u*_2_, and *u*_3_ is satisfied. The set of all the control variables (*u*_1_, *u*_2_, *u*_3_) ∈ *U* is also convex and closed by definition. The optimal system is bounded which determines the compactness needed for the existence of an optimal control. In addition the integrand in the functional (3), *A*_1_ *I*_*h*_ + *A*_2_ *I*_*b*_ + (1/2)(*C*_1_ *u*_1_^2^ + *C*_2_ *u*_2_^2^ + *C*_3_ *u*_3_^2^), is convex on the control set *U*. Also we can see that there exist a constant *ρ* > 1 and positive numbers *ω*_1_ and *ω*_2_ such that

(14) $$\begin{array}{c} J\left(u_{1},u_{2},u_{3}\right)\geq \omega_{1}{\left({\left|u_{1}\right|}^{2}+{\left|u_{2}\right|}^{2}+{\left|u_{3}\right|}^{2}\right)}^{\rho /2}-\omega_{2}, \end{array}$$

because the state variables are bounded, which complete the existence of an optimal control.

In order to derive the necessary conditions, we use Pontryagin's maximum principle [14] as follows. If (*x*, *u*) is an optimal solution of an optimal control problem, then there exists a nontrivial vector function *λ* = (*λ*_1_, *λ*_2_,…, *λ*_*n*_) satisfying the following equations:

(15) $$\begin{array}{c} \frac{dx}{dt}=\frac{\partial H\left(t,x,u,\lambda \right)}{\partial \lambda },\;\;0=\frac{\partial H\left(t,x,u,\lambda \right)}{\partial u},\\ \frac{d\lambda }{dt}=\frac{\partial H\left(t,x,u,\lambda \right)}{\partial x}. \end{array}$$

We now derive the necessary conditions that optimal control functions and corresponding states must satisfy. In the following theorem, we present the adjoint system and control characterization.

**Theorem 2**

Let *X*_h_*, *E*_h_*, *I*_h_*, *T*_h_*, *R*_h_*, *X*_b_*, *E*_b_*, and *I*_b_* be optimal state solutions with associated optimal control variables (*u*_1_*, *u*_2_*, and *u*_3_*) for the optimal control problem (1)–(3). Then there exist adjoint variables *λ*_*i*_, for *i* = 1,2,…, 8, satisfying

(16) dλ1dt=(λ1−λ2)(1−u1)(α1Ih+α2Ib)+λ1μh,dλ2dt=(λ2−λ3)ϕh+λ2μh, dλ3dt=(λ1−λ2)(1−u1)α1Xh+(λ3−λ4)ρh+λ3(βh+μh+u2)−λ5u2−A1, dλ4dt=(λ4−λ5)γh+λ4μh,dλ5dt=(λ5−λ1)ϵh+λ5μh,dλ6dt=(λ6−λ7)(1−u3)α3Ib+λ6μb,dλ7dt=(λ7−λ8)ϕb+λ7μb,dλ8dt=(λ6−λ7)(1−u3)α3Xb+λ8(βb+μb)−A2

with transversality conditions

(17) $$\begin{array}{c} \lambda_{i}\left(t_{\text{end}}\right)=0,\;i=1,2,\ldots ,8. \end{array}$$

Furthermore the control functions *u*_1_*, *u*_2_*, and *u*_3_* are given by

(18) u1∗=max⁡{min⁡{(λ2−λ1)α1Xh∗Ih∗c1,1},0},u2∗=max⁡{min⁡{(λ3−λ5)Ih∗c2,1},0},u3∗=max⁡{min⁡{(λ7−λ6)α3Xb∗Ib∗c3,1},0}.

**Proof**

To determine the adjoint equations and the transversality conditions, we use the Hamiltonian *H* in (12). The adjoint system results from Pontryagin's maximum principle [14]:

(19) $$\begin{array}{c} \frac{d\lambda_{1}}{dt}=-\frac{\partial H}{\partial X_{h}},\;\;\frac{d\lambda_{2}}{dt}=-\frac{\partial H}{\partial E_{h}},\;\;\frac{d\lambda_{3}}{dt}=-\frac{\partial H}{\partial I_{h}},\\ \frac{d\lambda_{4}}{dt}=-\frac{\partial H}{\partial T_{h}},\;\;\frac{d\lambda_{5}}{dt}=-\frac{\partial H}{\partial R_{h}},\;\;\frac{d\lambda_{6}}{dt}=-\frac{\partial H}{\partial X_{b}},\\ \frac{d\lambda_{7}}{dt}=-\frac{\partial H}{\partial E_{b}},\;\;\frac{d\lambda_{8}}{dt}=-\frac{\partial H}{\partial I_{b}} \end{array}$$

with *λ*_*i*_(*t*_end_) = 0, *i* = 1,2,…, 8.

To get the characterization of the optimal control given by (18), solving the equations

(20) $$\begin{array}{c} \frac{\partial H}{\partial u_{1}}=0,\;\;\frac{\partial H}{\partial u_{2}}=0,\;\;\frac{\partial H}{\partial u_{3}}=0, \end{array}$$

on the interior of the control set, and setting the property of the control space *U*, we can derive the desired characterization (18).

## 4. Numerical Results and Discussion

In this section, we present a semi-implicit finite difference method by discretizing the interval [*t*_0_, *t*_*f*_] at the points *t*_*i*_ = *t*_0_ + *il*, (*i* = 0,1,…, *n*), where *l* represents the time step such that *t*_*n*_ = *t*_*f*_. We define the state and adjoint variables *X*_*h*_, *E*_*h*_, *I*_*h*_, *T*_*h*_, *R*_*h*_, *X*_*b*_, *E*_*b*_, *I*_*b*_, *λ*_1_, *λ*_2_, *λ*_3_, *λ*_4_, *λ*_5_, *λ*_6_, *λ*_7_, and *λ*_8_ and the controls *u*_1_, *u*_2_, and *u*_3_ in terms of nodal points *X*_*h*_^*i*^, *E*_*h*_^*i*^, *I*_*h*_^*i*^, *T*_*h*_^*i*^, *R*_*h*_^*i*^, *X*_*b*_^*i*^, *E*_*b*_^*i*^, *I*_*b*_^*i*^, *λ*_1_^*i*^, *λ*_2_^*i*^, *λ*_3_^*i*^, *λ*_4_^*i*^, *λ*_5_^*i*^, *λ*_6_^*i*^, *λ*_7_^*i*^, *λ*_8_^*i*^, *u*_1_^*i*^, *u*_2_^*i*^, and *u*_3_^*i*^. By combination of forward and backward difference approximation, the method developed by [19] to adopt it in our case is given by

(21) Xhi+1−Xhil=Λ−μhXhi+1−(1−u1i)α1Xhi+1Ihi−α2Xhi+1Ibi+ϵhRhi,Ehi+1−Ehil=(1−u1i)α1Xhi+1Ihi+α2Xhi+1Ibi−(μh+ϕh)Ehi+1,Ihi+1−Ihil=ϕhEhi+1−(ρh+βh+μh+u2i)Ihi+1,Thi+1−Thil=ρhIhi+1−(γh+μh)Thi+1,Rhi+1−Rhil=γhTi+1+u2iIhi+1−(μh+ϵh)Rhi+1,Xbi+1−Xbil=π−μbXbi+1−(1−u3i)α3Xbi+1Ibi,Ebi+1−Ebil=(1−u3i)α3Xbi+1Ibi−(μb+ϕb)Ebi+1,Ibi+1−Ibil=ϕbEbi+1−(βb+μb)Ibi+1.

To approximate the time derivative of the adjoint variables by the first-ordered backward difference, we use the appropriate scheme as follows:

(22) λ1n−i−λ1n−i−1l=(λ1n−i−1−λ2n−i)(1−u1i)(α1Ihi+1+α2Ibi+1) +λ1n−i−1μh,λ2n−i−λ2n−i−1l=(λ2n−i−1−λ3n−i)ϕh+λ2n−i−1μh,λ3n−i−λ3n−i−1l=(λ1n−i−1−λ2n−i−1)(1−u1i)α1Xhi+1 +λ3n−i−1(ρh+βh+μh+u2i) −λ5n−iu2i−λ4n−iρh−A1,λ4n−i−λ4n−i−1l=(λ4n−i−1−λ5n−i)γh+λ4n−i−1μh,λ5n−i−λ5n−i−1l=(λ5n−i−1−λ1n−i−1)ϵh+λ5n−i−1μh,λ6n−i−λ6n−i−1l=(λ6n−i−1−λ7n−i)(1−u3i)α3Ibi+1+λ6n−i−1μb,λ7n−i−λ7n−i−1l=(λ7n−i−1−λ8n−i)ϕb+λ7n−i−1μb,λ8n−i−λ8n−i−1l=(λ6n−i−1−λ7n−i−1)(1−u3i)α3Xbi+1 +λ8n−i−1(βb+μb)−A2.

The algorithm that describes the approximation method for obtaining the optimal control is as follows.

**Algorithm 3**

Consider the following.

*Step 1*. Consider *X*_*h*_(0) = *X*_*h*0_, *E*_*h*_(0) = *E*_*h*0_, *I*_*h*_(0) = *I*_*h*0_, *T*_*h*_(0) = *T*_*h*0_, *R*_*h*_(0) = *R*_*h*0_, *X*_*b*_(0) = *X*_*b*0_, *E*_*b*_(0) = *E*_*b*0_, *I*_*b*_(0) = *I*_*b*0_, *u*_1_(0) = *u*_2_(0) = *u*_3_(0) = 0, and *λ*_*i*_(*t*_*f*_) = 0, *i* = 1,…, 8.

*Step 2*. For *i* = 1,…, *n* − 1, do

(23) Xhi+1=Xhi+l[Λ+ϵhRhi]1+l[μh+(1−u1i)α1Ihi+α2Ibi],Ehi+1=Ehi+l[(1−u1i)α1Xhi+1Ihi+α2Xhi+1Ibi]1+l[μh+ϕh],Ihi+1=Ihi+lϕhEhi+11+l[ρh+βh+μh+u2i],Thi+1=Thi+lρhIhi+11+l[γh+μh],Rhi+1=Rhi+l[γThi+1+u2iIhi+1]1+l[μh+ϵh],Xbi+1=Xbi+lπ1+l[μb+(1−u3i)α3Ibi],Ebi+1=Ebi+l(1−u3i)α3Xbi+1Ibi1+l[μb+ϕb],Ibi+1=Ibi+lϕbEbi+11+l[βb+μb],λ1n−i−1=λ1n−i+lλ2n−i(1−u1i)[α1Ihi+1+α2Ihi+1]1+l[(1−u1i)α1Ihi+1+α2Ihi+1+μh],λ2n−i−1=λ3n−i+lλ3n−iϕh1+lλ2n−i−1[ϕh+μh],λ3n−i−1=λ3n−i+l[(λ2n−i−1−λ1n−i−1)(1−u1i)α1Xhi+1+ρhλ4n−i+u2iλ5n−i+A1] ×(1+l[ρh+βh+μh+u2i])−1,λ4n−i−1=λ4n−i−lγhλn−i1−l[γh+μh],λ5n−i−1=λ5n−i+lϵhλ1n−i−11+l[ϵh+μh],λ6n−i−1=λ6n−i+l(1−u3i)Ibi+1λ7n−i1+l[μb+(1−u3i)α3Ibi+1],λ7n−i−1=λ7n−i+lϕbλ8n−i1+l[ϕb+μb],λ8n−i−1=λ8n−i+l[(λ7n−i−1−λ6n−i−1)(1−u3i)α3Xbi+1+A2]1+l[βb+μb],u1i+1=max⁡{min⁡{(λ2n−i−1−λ1n−i−1)α1Xhi+1Ihi+1c1,1},0},u2i+1=max⁡{min⁡{(λ3n−i−1−λ5n−i−1)Ihi+1c2,1},0},u3i+1=max⁡{min⁡{(λ7n−i−1−λ6n−i−1)α3Xbi+1Ibi+1c3,1},0},

end for.

*Step 3*. For *i* = 1,…, *n* − 1, write *X*_h_*(*t*_*i*_) = *X*_*h*_^*i*^, *E*_h_*(*t*_*i*_) = *E*_*h*_^*i*^, *I*_h_*(*t*_*i*_) = *I*_*h*_^*i*^, *T*_h_*(*t*_*i*_) = *T*_*h*_^*i*^, *R*_h_*(*t*_*i*_) = *R*_*h*_^*i*^, *X*_b_*(*t*_*i*_) = *X*_*b*_^*i*^, *E*_b_*(*t*_*i*_) = *E*_*b*_^*i*^, *I*_b_*(*t*_*i*_) = *I*_*b*_^*i*^, *u*_1_*(*t*_*i*_) = *u*_1_^*i*^, *u*_2_*(*t*_*i*_) = *u*_2_^*i*^, and *u*_3_*(*t*_*i*_) = *u*_3_^*i*^ end for.

**Figure 1:**
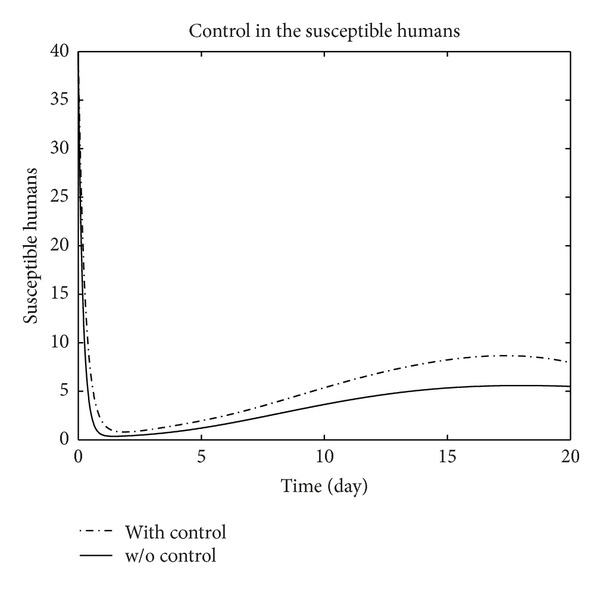
The plot represents the population of susceptible humans with and without control.

**Figure 2:**
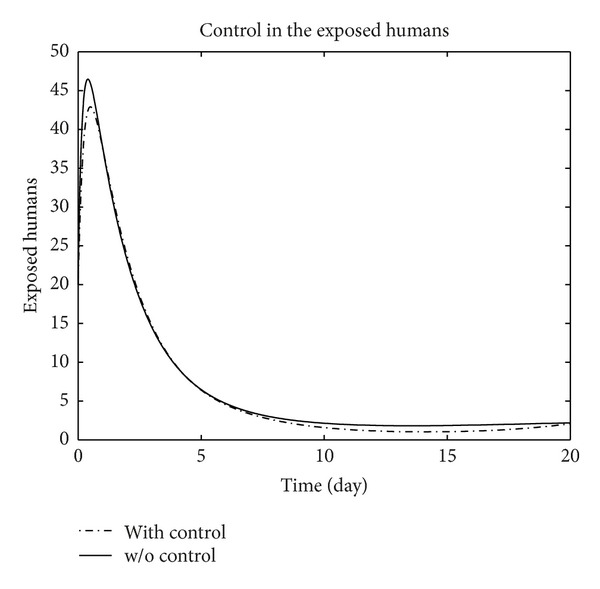
The plot represents the population of exposed humans with and without control.

**Figure 3:**
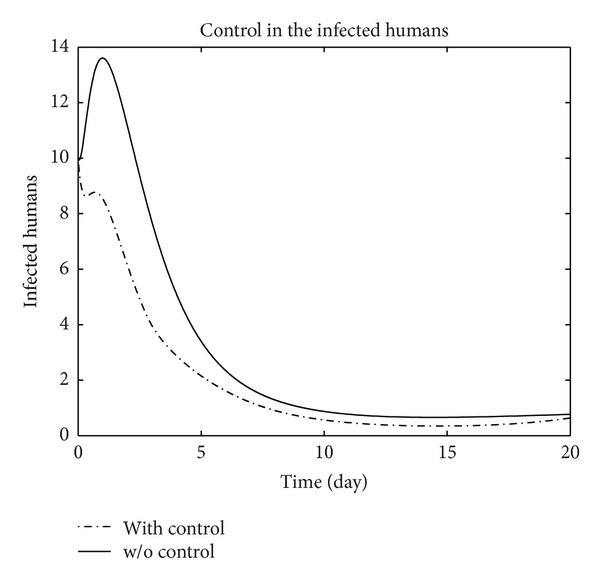
The plot represents the population of infected humans with and without control.

**Figure 4:**
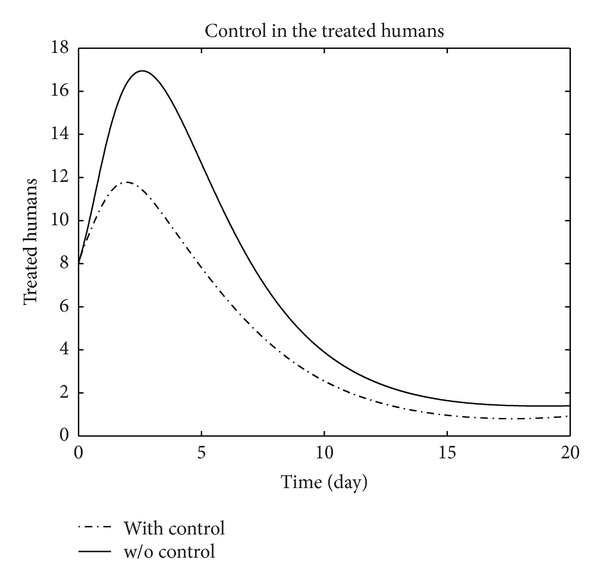
The plot represents the population of treated humans with and without control.

**Figure 5:**
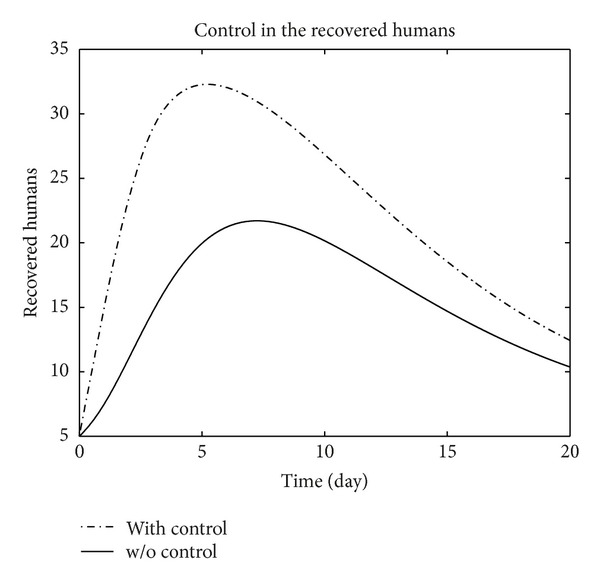
The plot represents the population of recovered humans with and without control.

**Figure 6:**
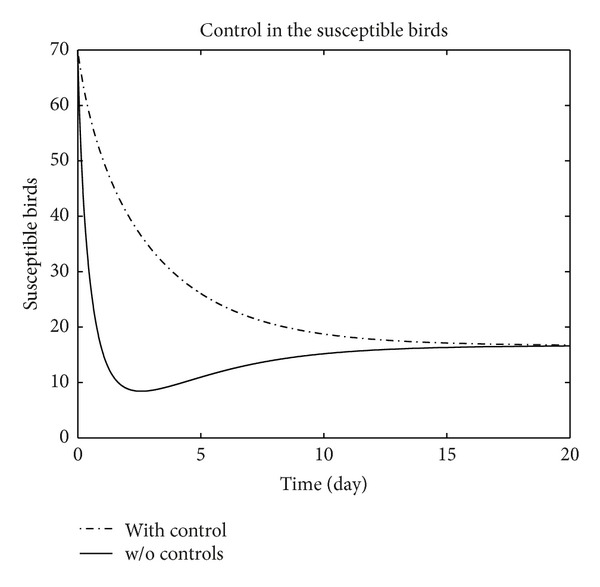
The plot represents the population of susceptible birds with and without control.

**Figure 7:**
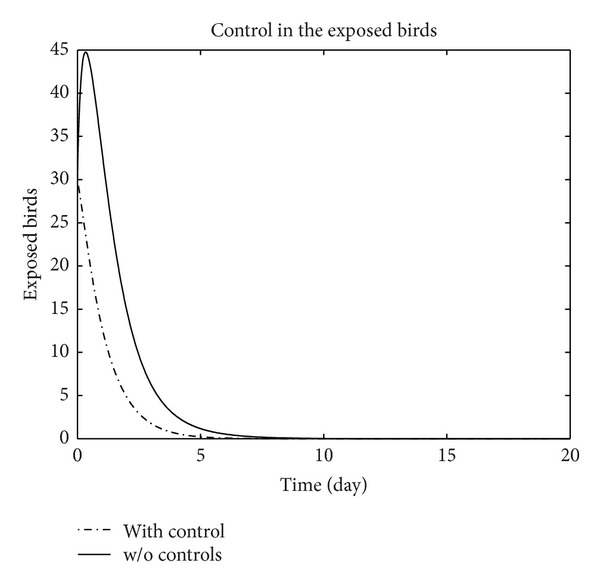
The plot represents the population of exposed birds with and without control.

**Figure 8:**
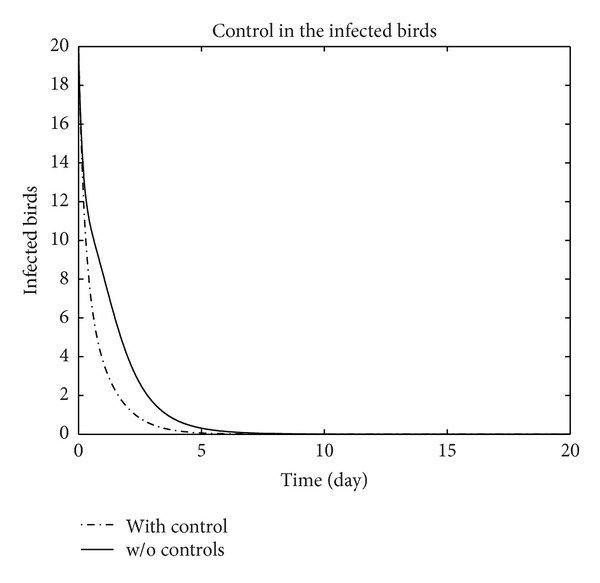
The plot represents the population of infected birds with and without control.

**Figure 9:**
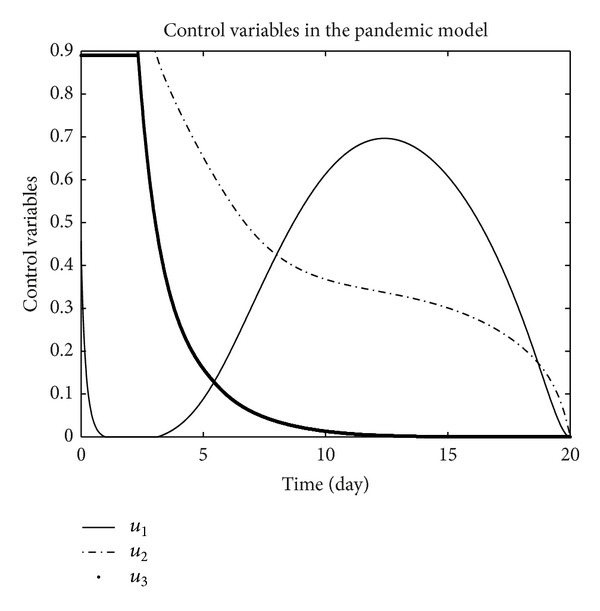
The plot represents the control variables *u*_1_, *u*_2_, and *u*_3_.

We have plotted susceptible, exposed, infected, treated, and recovered population of humans and susceptible, exposed, and infected population of birds with and without control by considering parameter values given in Table 1, with initial values *X*_*h*_(0) = 40, *E*_*h*_(0) = 20, *I*_*h*_(0) = 10, *T*_*h*_(0) = 8, *R*_*h*_(0) = 5, *X*_*b*_(0) = 70, *E*_*b*_(0) = 30, and *I*_*b*_(0) = 20. In each of the given figures the undashed and the dashed lines represent the individuals without and with control, respectively. The weight constant of the objective functional *A*_1_ = 0.035, *A*_2_ = 0.0015, *C*_1_ = 0.05, *C*_2_ = 0.08, and *C*_3_ = 0.002 is considered for numerical simulation.  Figure 1 shows that the population of the susceptible humans increased after control, in Figures 2 and 3 we see that the number of the exposed and infected humans with control decreased more sharply than that without control, Figure 4 shows that per day clinically reported humans decreased after control, Figure 5 shows that the number of recovered human individuals increased after control, Figure 6 shows that the birds population increased after control, and in Figures 7 and 8 we see that the number of the exposed and infected birds decreased after control.  Figure 9 shows the graphs of control variables.

## 5. Conclusion

An optimal control problem of the transmission dynamics of the human-avian influenza disease has been presented. We sought to determine three types of control functions associated with isolation and antiviral treatment of the clinically infectious individuals and elimination of the infected birds. Our control plots indicated that the number of exposed, infected, and hospitalized humans and the number of exposed, infected birds decreased in the optimal system. We developed the necessary conditions for the existence of the optimal control by using Pontryagin's maximum principle. Using the state and adjoint system together with the characterization of the optimal control, we solved the problem numerically by an efficient numerical method based on optimal control with the estimated parameter values based on avian influenza. The results show that the control practices are very effective in reducing the incidence of infectious population.

## Figures and Tables

**Table 1:** Parameter values used for numerical simulations.

Notation	Parameters definition	Value
Λ	Recruitment rate of humans	1.7/day
*α*_1_	Effective contact rate between *X*_*h*_ and *I*_*h*_	0.3
*α*_2_	Effective contact rate between *X*_*h*_ and *I*_*b*_	0.15
*α*_3_	Effective contact rate between *X*_*b*_ and *I*_*b*_	0.12
*π*	Recruitment rate of birds	5/day
*ϵ*_*h*_	Rate of immunity loss	0.005
*μ*_*h*_	Natural death rate of humans	0.1/day
*μ*_*b*_	Natural death rate of birds	0.3/day
*γ*_*h*_	Recovery due to treatment	0.3
*ρ*_*h*_	Treatment rate	0.75
*β*_*h*_	Disease induced death rate in humans	0.4/day
*β*_*b*_	Disease induced death rate in birds	3.7/day
*ϕ*_*h*_	Progression rate from *E*_*h*_ to *I*_*h*_	0.45
*ϕ*_*b*_	Progression rate from *E*_*b*_ to *I*_*b*_	0.85
